# Supplementary material for: The New Di-Gold Metallotweezer Based on an Alkynylpyridine System
Source: Molecules. 2022 Jun 9;27(12):3699. doi: 10.3390/molecules27123699 (PMC9227567; doi:10.3390/molecules27123699)
Supplement: Supplementary file 1 [file molecules-27-03699-s001.zip › molecules-1740997-supplementary.pdf]

Supporting information for:

1. Spectroscopic data **S1**

1.1. ^{1}H and ^{13}C NMR spectra of **2** S1

1.1. ^{1}H and ^{13}C NMR spectra of **3@2** S2

2. Photophysical analysis **S3**

2.1. UV-visible absorption spectra S3

2.2. Emission spectra S3

3. Titration experiments **S4-S19**

3.1. ^{1}H NMR titrations **S4**

4. High Resolution mass spectra **S20**

5. Dosy experiments **S21**

6. IR **S22**

1. Spectroscopic data

1.1. ^1H and ^{13}C NMR spectra of 2

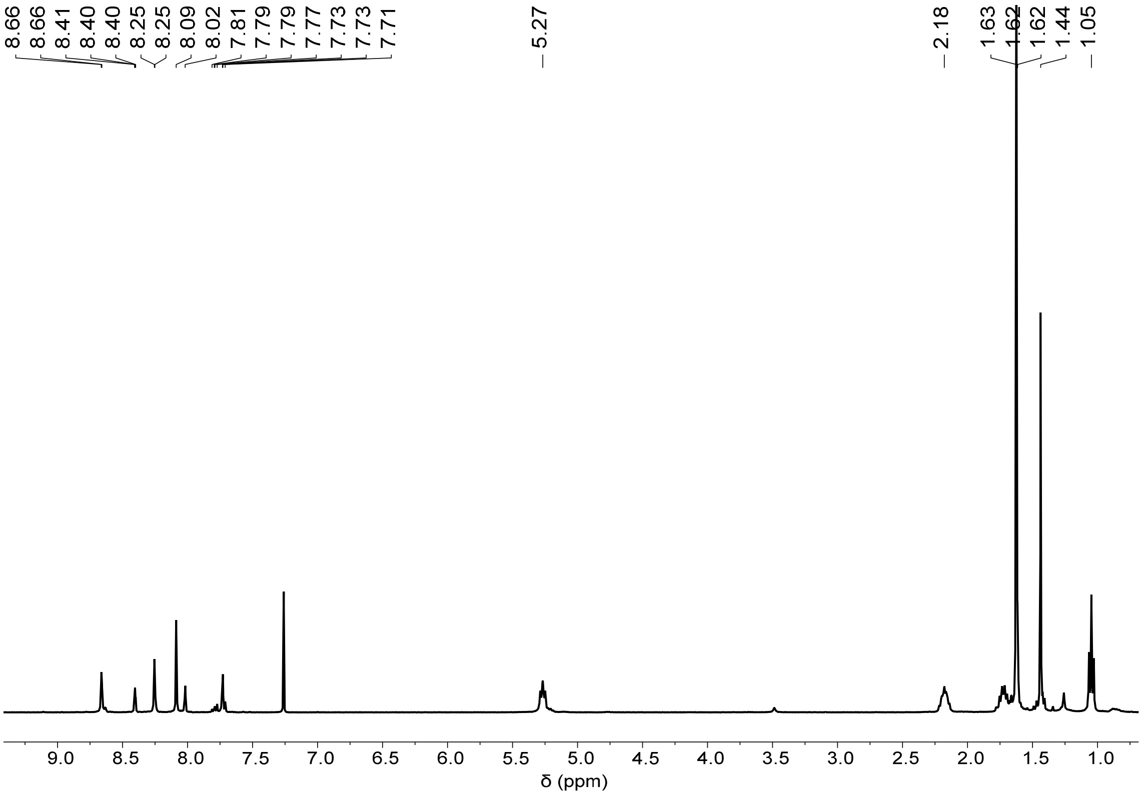

Figure S1. ^1H NMR spectrum of **2** in $CDCl_3$

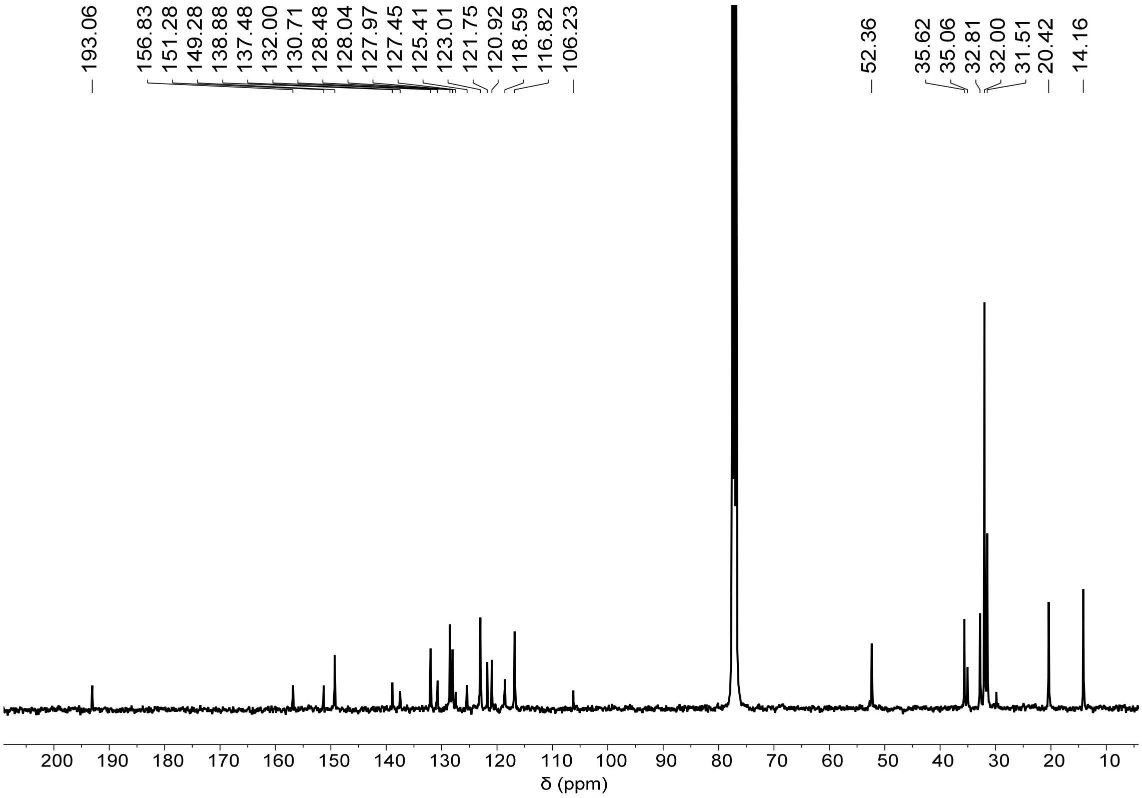

Figure S2. ^{13}C NMR spectrum of **2** in $CDCl_3$

1.2. ^{1}H and ^{13}C NMR spectra of 3@2

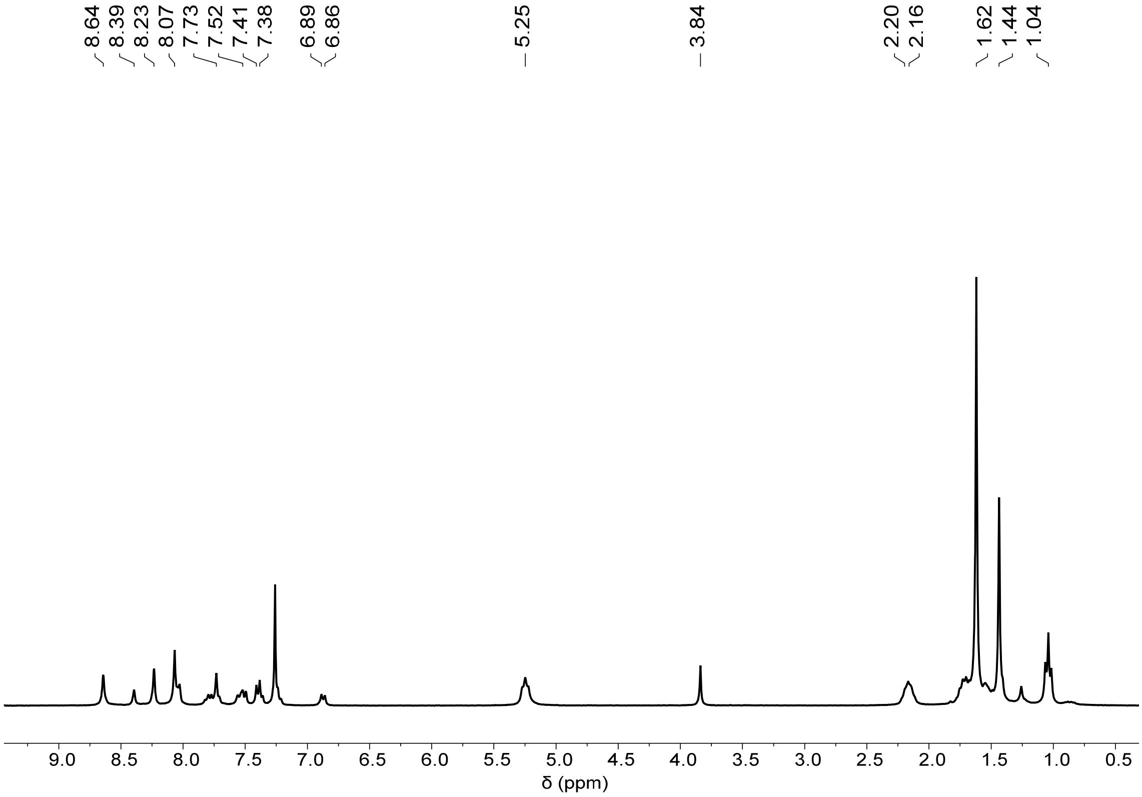

Figure S3. ^{1}H NMR spectrum of **3@2** in $CDCl_3$

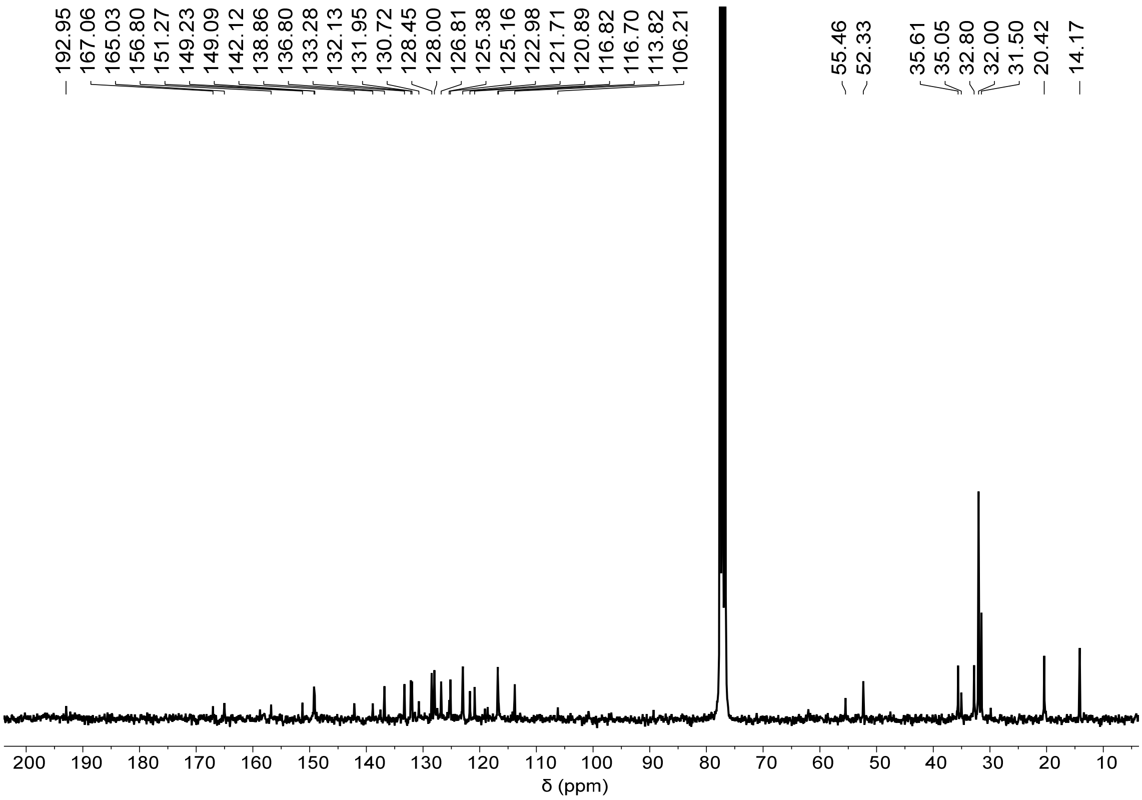

Figure S4. ^{13}C NMR spectrum of **3@2** in $CDCl_3$

2. Photophysical analysis

2.1. UV-visible absorption spectra

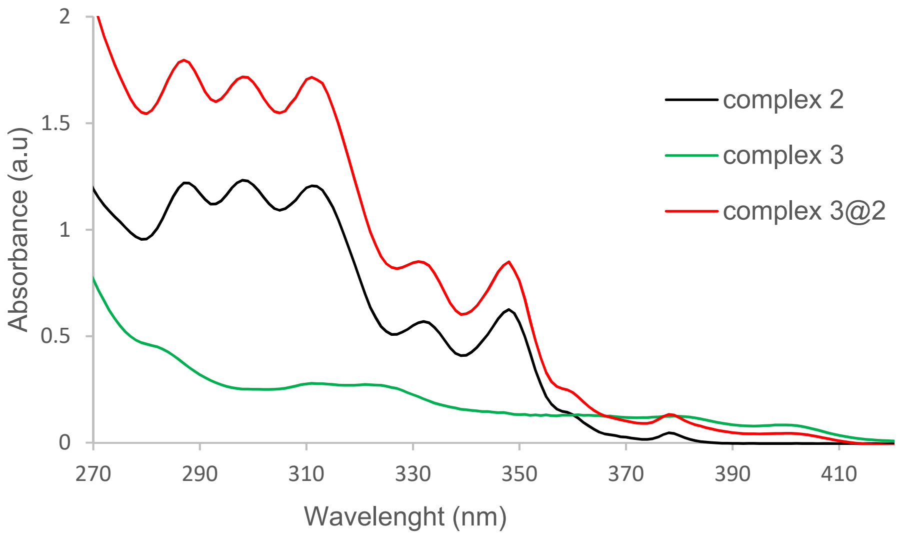

Figure S5. UV-visible absorption spectra of complexes **2** and **3@2**, recorded in CH_2Cl_2 at a concentration of 10^{-5} M, under aerobic conditions at room temperature.

3.2. Emission spectra

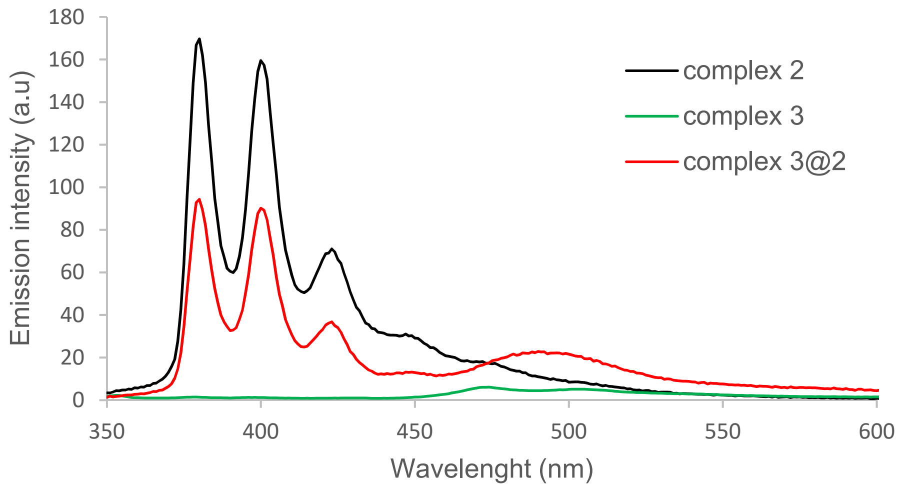

Figure S6. Emission spectra of complexes **2** and **3@2** at 320 nm in CH_2Cl_2 at a concentration of 10^{-5} M, under aerobic conditions at room temperature.

3. ^{1}H NMR titration experiments

3.1. ^{1}H NMR titrations

The recognition capability of complex **2** (host) was studied by ^{1}H NMR titration experiments, by adding increasing amounts of different guest as pyrene, 1-pyrenyl-methanol, triphenylene, perylene, 3-perylenyl-methanol, coronene or electron poor organic substrates as N,N'-dimethyl-naphthalenetetracarboxy diimide (NTCDI), 2,4,7-trinitro-9-fluorenone (TNFLU), and a planar Pt(II) or Au(III) complex containing a CNC pincer ligand to a solution of complex **2**. The experiment was carried out in $CDCl_3$, at constant concentrations of the host (1 mM). Two solutions were prepared: solution A (only containing host at 1 mM) and solution B (containing host at 1 mM and guest at different mM). The addition of increasing amounts of solution B to solution A produced perturbations on the signal due to the proton of the triphenylene core of the host. The association constants were calculated by nonlinear least-square analysis, by using the BindFitv0.5 program.

Titration of 2 with pyrene

Table S1. Data values from the titration study of **2** with pyrene

**[2] M**	[pyrene] M	δ_{CH}	δ_{NCH2}	equiv. pyrene
0,00100185	0	8,66	5,27	0
0,00100185	0,00209185	8,66	5,27	2,1
0,00100185	0,00402875	8,66	5,26	4,0
0,00100185	0,0058273	8,65	5,26	5,8
0,00100185	0,00750181	8,65	5,26	7,5
0,00100185	0,00906469	8,65	5,26	9,0
0,00100185	0,01052674	8,65	5,25	10,5
0,00100185	0,0118974	8,64	5,25	11,9
0,00100185	0,01439686	8,64	5,25	14,4
0,00100185	0,0166186	8,64	5,25	16,6
0,00100185	0,02039555	8,64	5,25	20,3
0,00100185	0,02348578	8,64	5,24	23,4
0,00100185	0,02719407	8,63	5,24	27,1

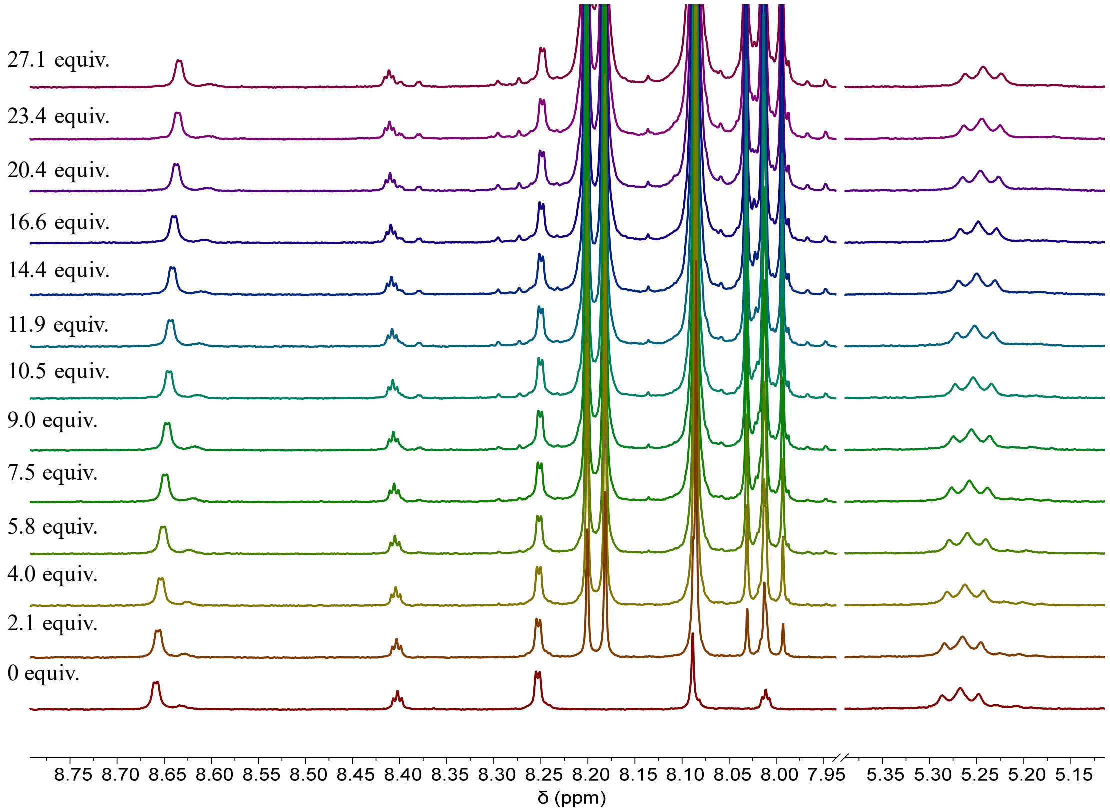

Figure S7. Selected region and spectra of the titration of complex **2** with pyrene.

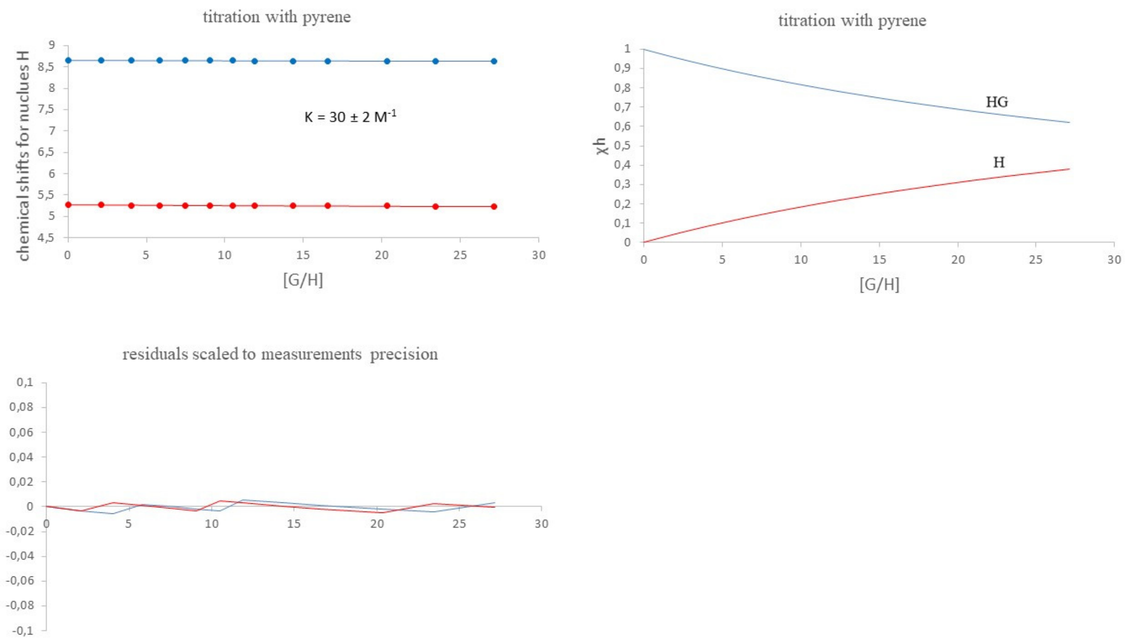

Figure S8. Non-linear least-squares fitting of the chemical shift changes of H during titration experiments of **2** with pyrene. The Figure on the left represents the speciation profiles.

Titration of 2 with 1-pyrenyl-methanol

Table S2. Data values from the titration study of **2** with 1-pyrenyl-methanol

**[2] M**	[1-pyrenyl-methanol] M	δ_{CH}	δ_{NCH2}	equiv. 1-pyrenyl-methanol
0,00106786	0	8,66	5,27	0
0,00106786	0,00092726	8,66	5,27	0,9
0,00106786	0,00178584	8,66	5,26	1,7
0,00106786	0,00258309	8,66	5,26	2,4
0,00106786	0,00332536	8,65	5,26	3,1
0,00106786	0,00401814	8,65	5,25	3,8
0,00106786	0,00497484	8,65	5,25	4,7
0,00106786	0,00611717	8,65	5,25	5,7
0,00106786	0,00713078	8,65	5,24	6,7
0,00106786	0,00824776	8,64	5,24	7,7
0,00106786	0,01009207	8,64	5,24	9,4
0,00106786	0,01205442	8,64	5,24	11,3

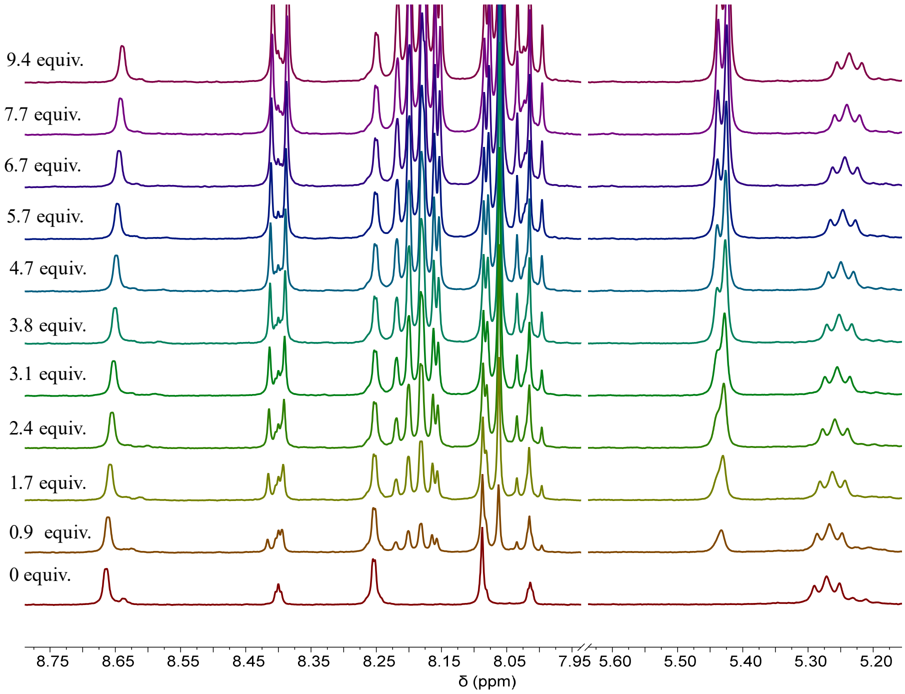

Figure S9. Selected region and spectra of the titration of complex **2** with 1-pyrenyl-methanol.

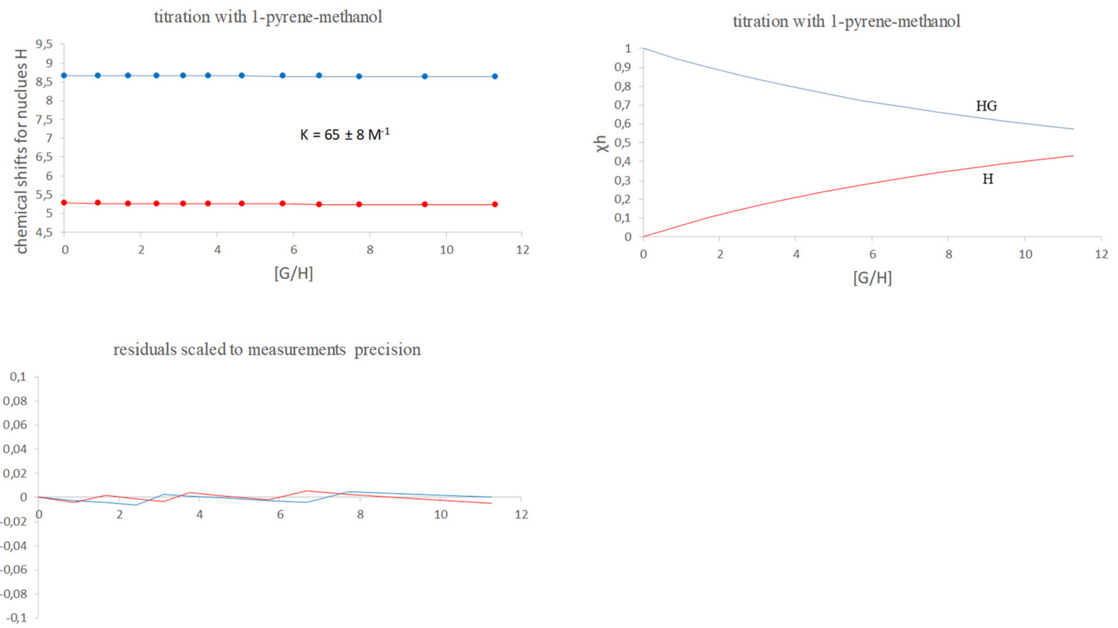

Figure S10. Non-linear least-squares fitting of the chemical shift changes of H during titration experiments of **2** with 1-pyrenyl-methanol. The Figure on the left represents the speciation profiles.

Titration of 2 with triphenylene

Table S3. Data values from the titration study of **2** with triphenylene

**[2] M**	[triphenylene] M	δ_{NCH2}	equiv. triphenylene
0,00110669	0	5,27	0
0,00110669	0,00047171	5,27	0,4
0,00110669	0,00090849	5,27	0,8
0,00110669	0,00169166	5,26	1,5
0,00110669	0,00237379	5,26	2,1
0,00110669	0,0032465	5,26	2,9
0,00110669	0,00440266	5,26	4,0
0,00110669	0,00600713	5,26	5,4
0,00110669	0,0072381	5,25	6,5
0,00110669	0,00817638	5,25	7,4

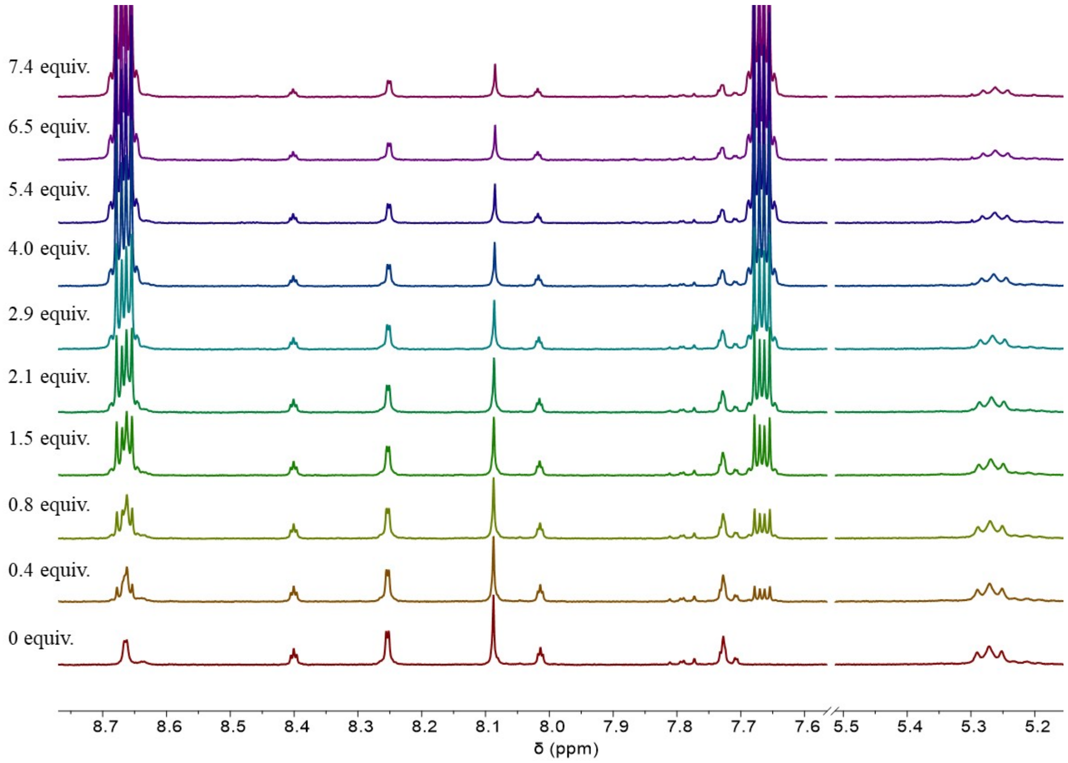

Figure S11. Selected region and spectra of the titration of complex **2** with triphenylene.

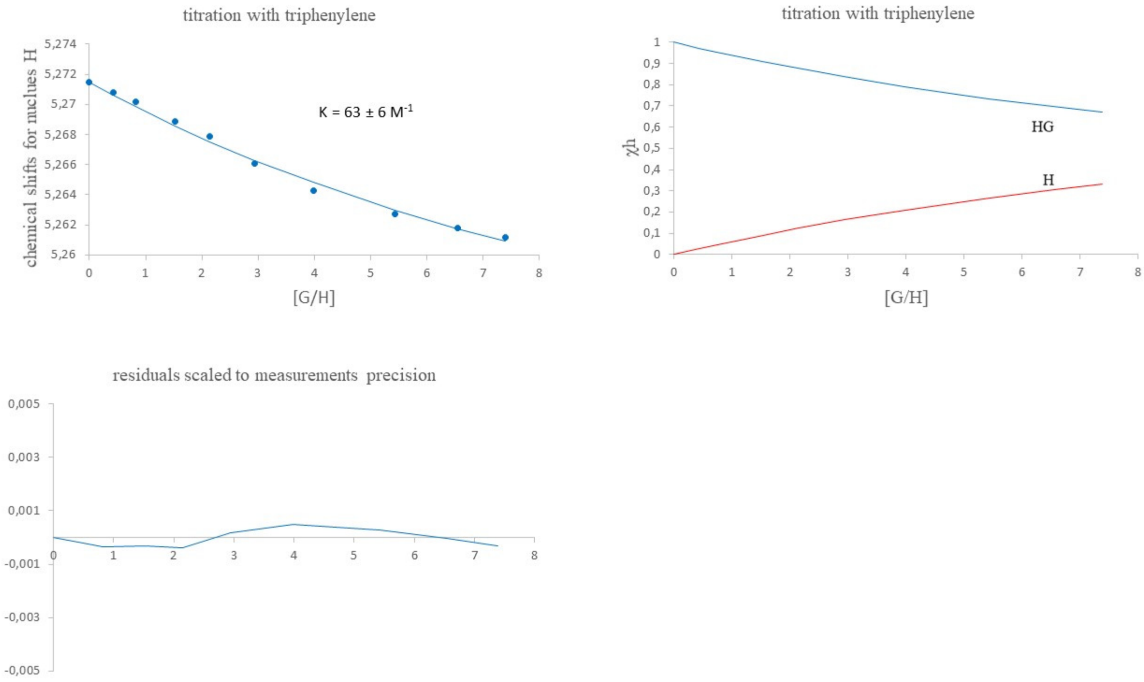

Figure S12. Non-linear least-squares fitting of the chemical shift changes of H during titration experiments of **2** with triphenylene. The Figure on the left represents the speciation profiles.

Titration of 2 with perylene

Table S4. Data values from the titration study of **2** with perylene

**[2] M**	[perylene] M	δ_{CH}	δ_{NCH2}	equiv. perylene
0,00106786	0	8,66	5,27	0
0,00106786	0,00039632	8,66	5,27	0,4
0,00106786	0,00076329	8,66	5,27	0,7
0,00106786	0,00110404	8,66	5,26	1,0
0,00106786	0,0017174	8,65	5,26	1,6
0,00106786	0,00249803	8,65	5,26	2,3
0,00106786	0,00352518	8,65	5,26	3,3
0,00106786	0,00493751	8,65	5,26	4,6
0,00106786	0,00586283	8,65	5,25	5,5
0,00106786	0,00686958	8,64	5,25	6,4

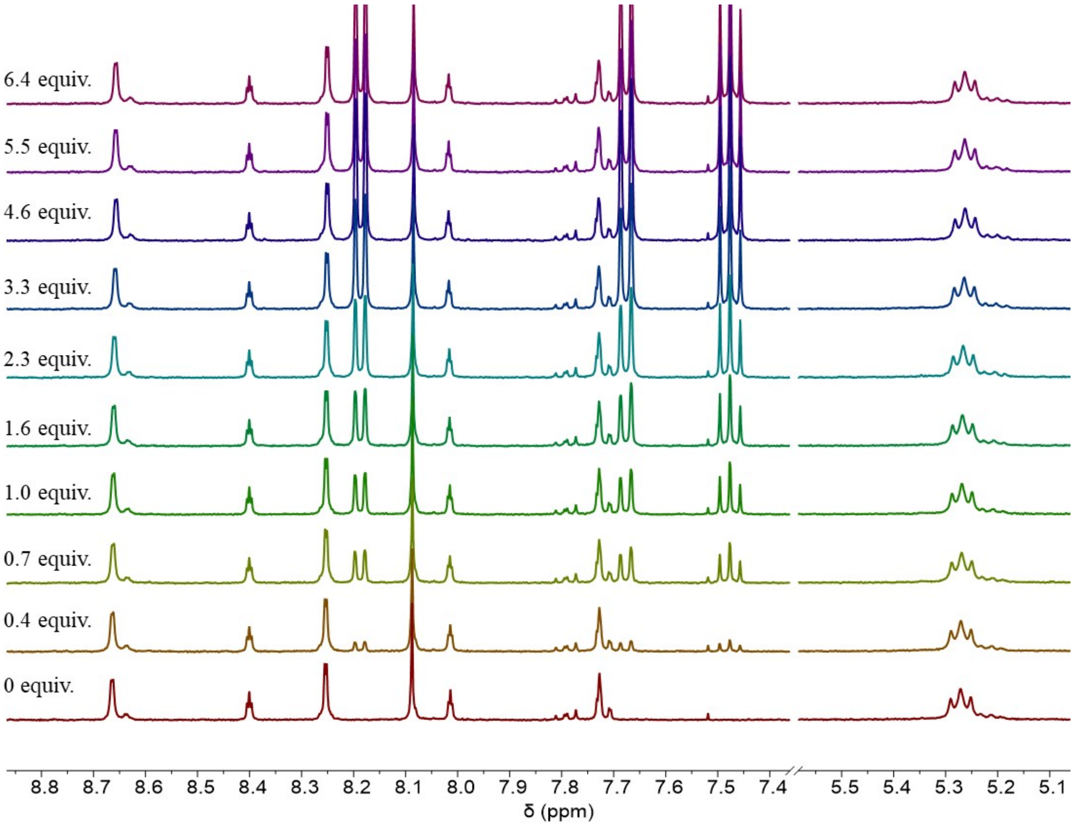

Figure S13. Selected region and spectra of the titration of complex **2** with perylene.

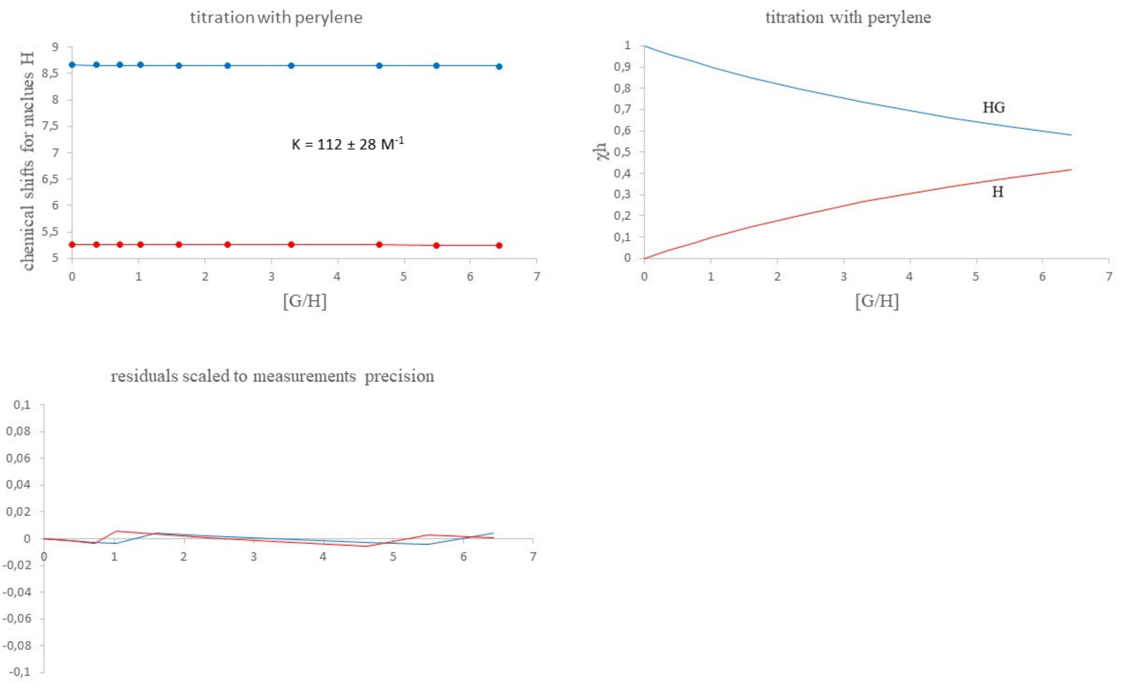

Figure S14. Non-linear least-squares fitting of the chemical shift changes of H during titration experiments of **2** with perylene. The Figure on the left represents the speciation profiles.

Titration of 2 with 3-perylenyl-methanol

Table S5. Data values from the titration study of **2** with 3-perylenyl-methanol

**[2] M**	[3-perylenyl-methanol] M	δc_H	δ_{NCH2}	equiv. 3- perylenyl-methanol
0,00104845	0	8,66	5,27	0
0,00104845	0,00036783	8,66	5,26	0,4
0,00104845	0,00070842	8,65	5,26	0,7
0,00104845	0,00131912	8,65	5,25	1,3
0,00104845	0,00185102	8,64	5,24	1,8
0,00104845	0,00231845	8,64	5,23	2,2
0,00104845	0,00292222	8,63	5,23	2,8
0,00104845	0,00358636	8,63	5,22	3,4
0,00104845	0,00412974	8,62	5,22	3,9

0,00104845	0,00468422	8,62	5,21	4,5
0,00104845	0,0056441	8,61	5,21	5,4
0,00104845	0,00637574	8,61	5,21	6,1

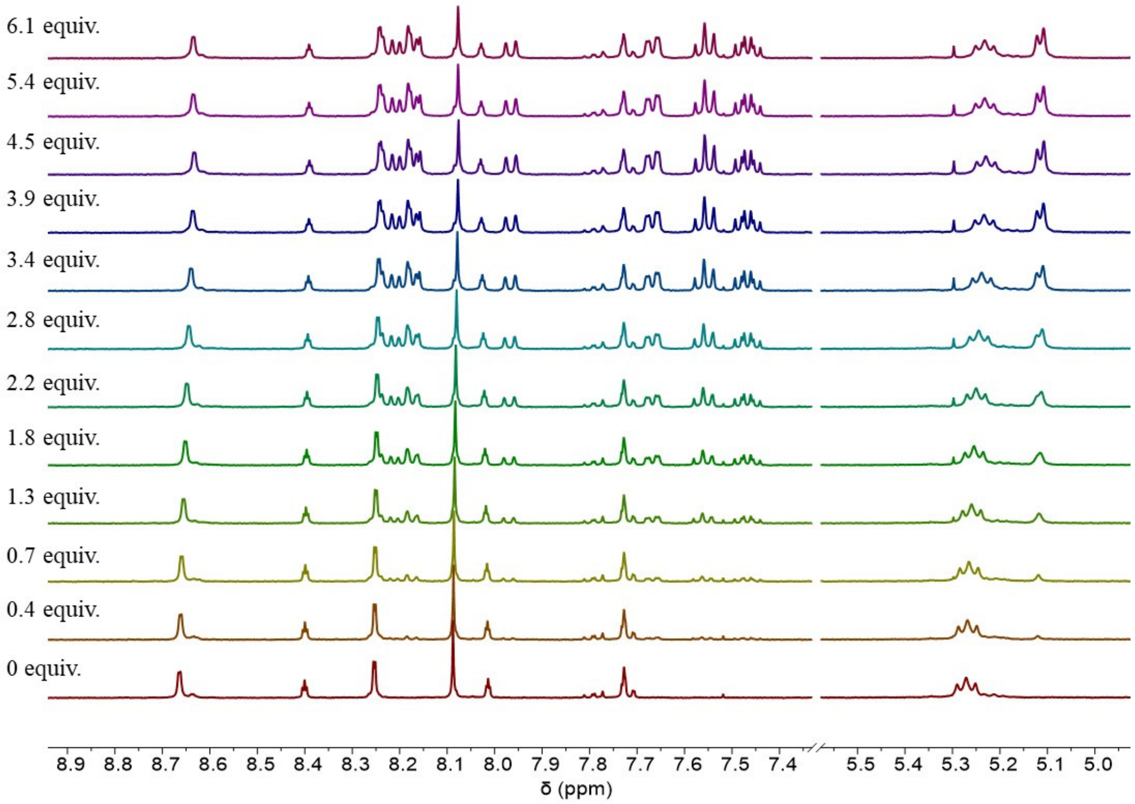

Figure S15. Selected region and spectra of the titration of complex **2** with 3-perylenyl-methanol.

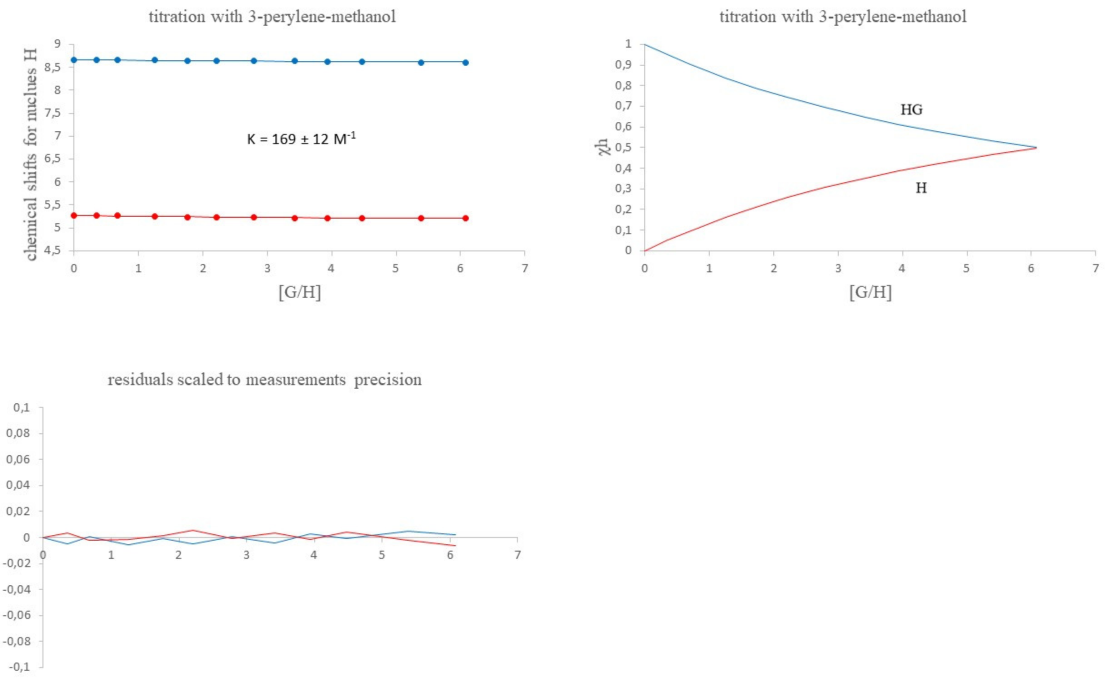

Figure S16. Non-linear least-squares fitting of the chemical shift changes of H during titration experiments of **2** with 3-perylenyl-methanol. The Figure on the left represents the speciation profiles.

Titration of 2 with coronene

Table S6. Data values from the titration study of **2** with coronene

**[2] M**	[coronene] M	δ_{CH}	δ_{NCH2}	equiv. coronene
0,489274406	0	8,66	5,27	0
0,489274406	0,18439411	8,66	5,27	0,4
0,489274406	0,3551294	8,66	5,26	0,7
0,489274406	0,51366931	8,65	5,26	1,0
0,489274406	0,79904115	8,65	5,26	1,6
0,489274406	1,04874151	8,65	5,25	2,1
0,489274406	1,32015494	8,64	5,25	2,7
0,489274406	1,55489089	8,64	5,24	3,2
0,489274406	2,00689405	8,64	5,24	4,1
0,489274406	2,39712345	8,63	5,24	4,9

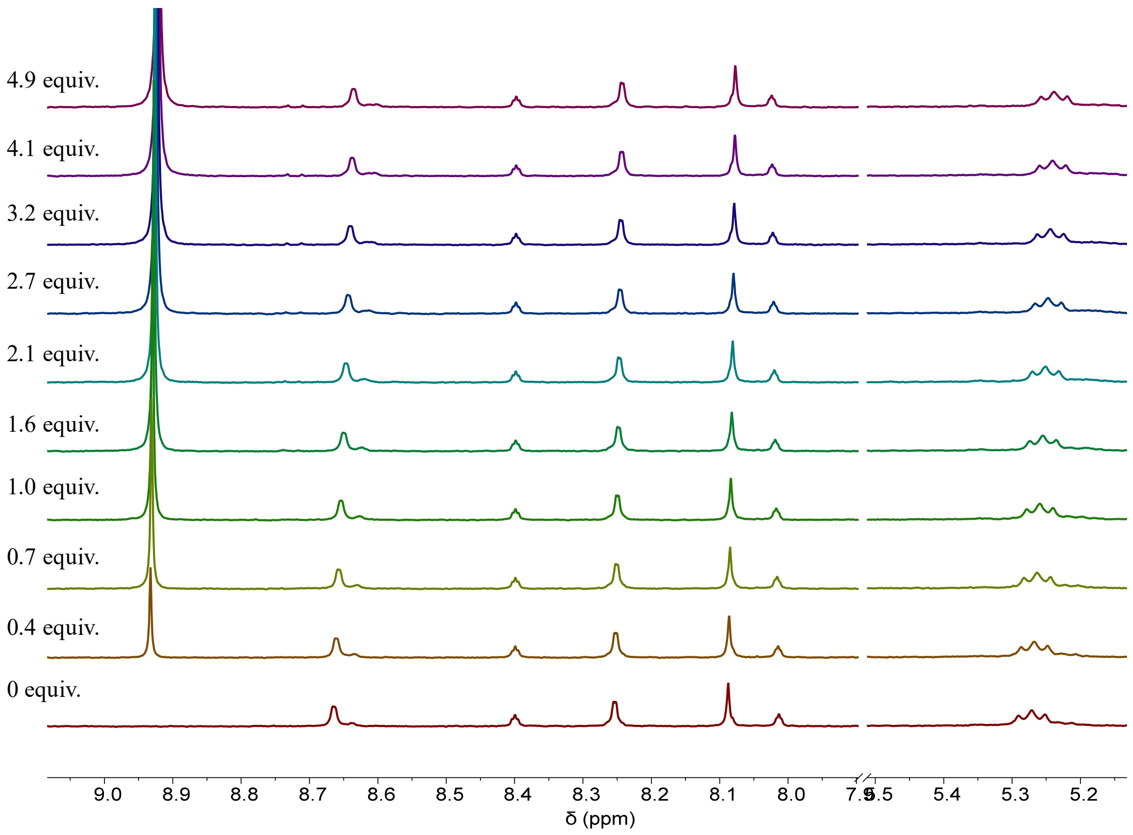

Figure S17. Selected region and spectra of the titration of complex **2** with coronene.

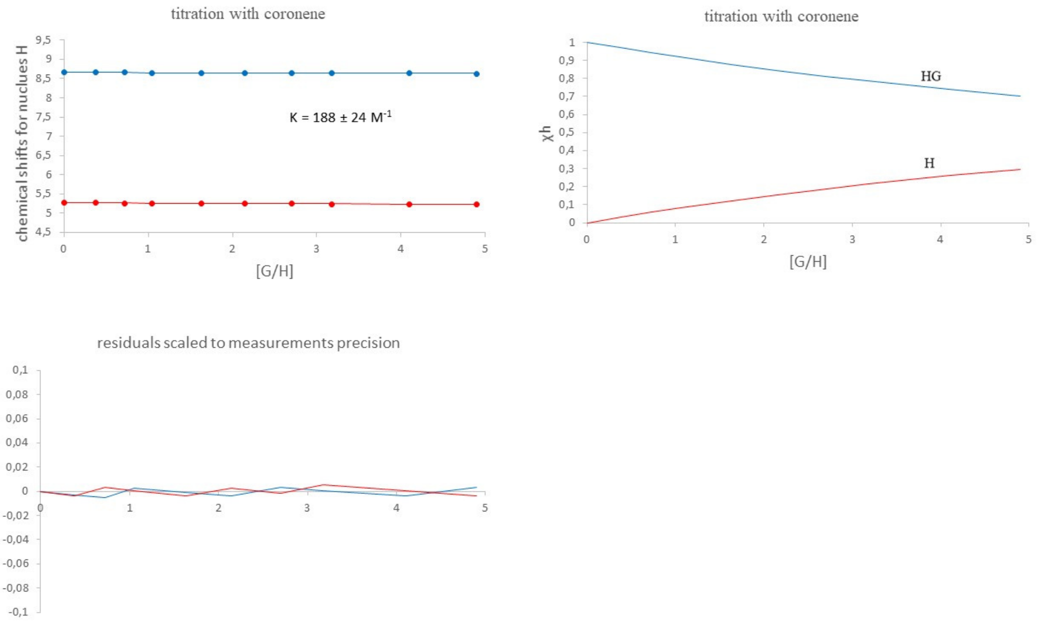

Figure S18. Non-linear least-squares fitting of the chemical shift changes of H during titration experiments of **2** with coronene. The Figure on the left represents the speciation profiles.

Titration of 2 with NTCDI

Table S7. Data values from the titration study of **2** with NTCDI

**[2]** M	[NTCDI] M	δc_H	δc_H	δc_H	δ_{NCH2}	equiv. NTCDI
0,00104845	0	8,663	8,4014	8,0887	5,2682	0
0,00104845	0,00049668	8,6584	8,3989	8,0851	5,2667	0,5
0,00104845	0,00095657	8,6539	8,3978	8,0806	5,2667	0,9
0,00104845	0,00138362	8,6503	8,3943	8,0756	5,2643	1,3
0,00104845	0,00178121	8,6479	8,3919	8,0721	5,2627	1,7
0,00104845	0,00249944	8,6408	8,3883	8,0651	5,2572	2,4
0,00104845	0,00313061	8,6348	8,3834	8,0591	5,2555	3,0
0,00104845	0,00418824	8,6278	8,3778	8,0496	5,2515	4,0
0,00104845	0,00573944	8,6208	8,3753	8,0415	5,2491	5,5
0,00104845	0,00724982	8,6194	8,3743	8,039	5,246	6,9
0,00104845	0,00860917	8,6183	8,3743	8,039	5,245	8,2

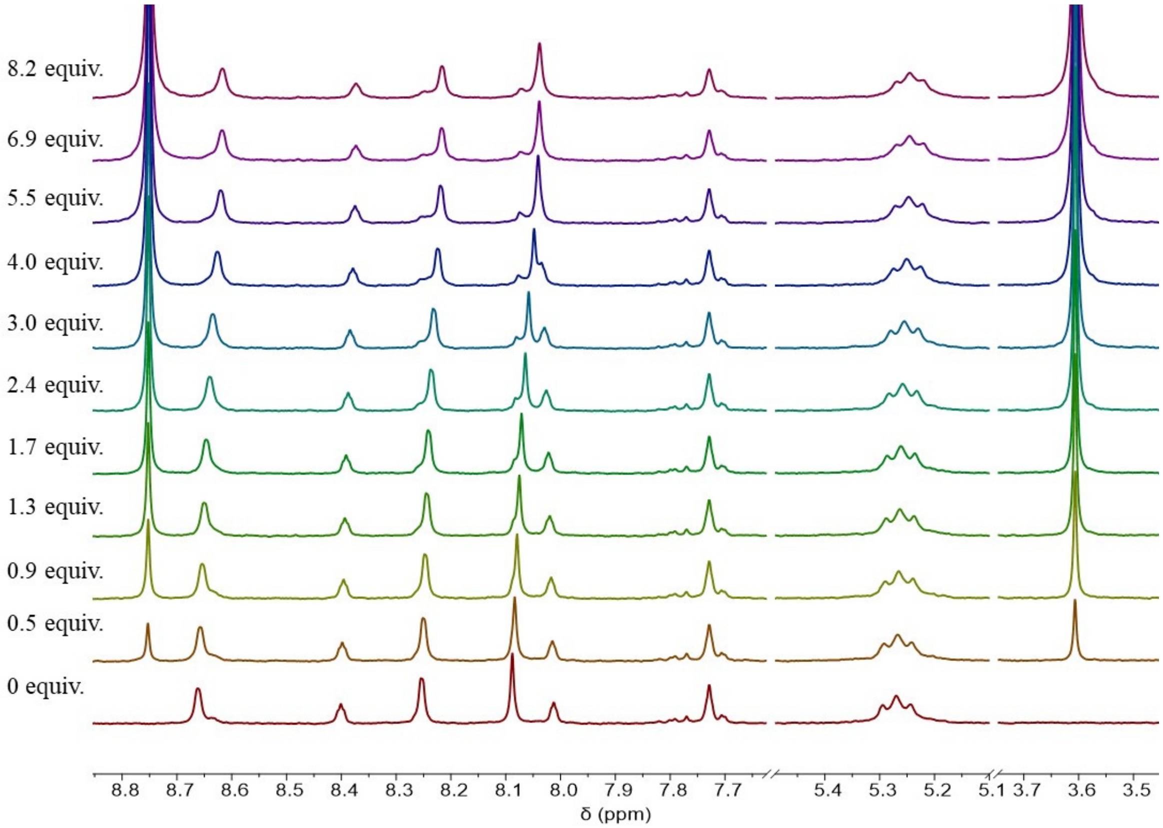

Figure S19. Selected region and spectra of the titration of complex **2** with NTCDI.

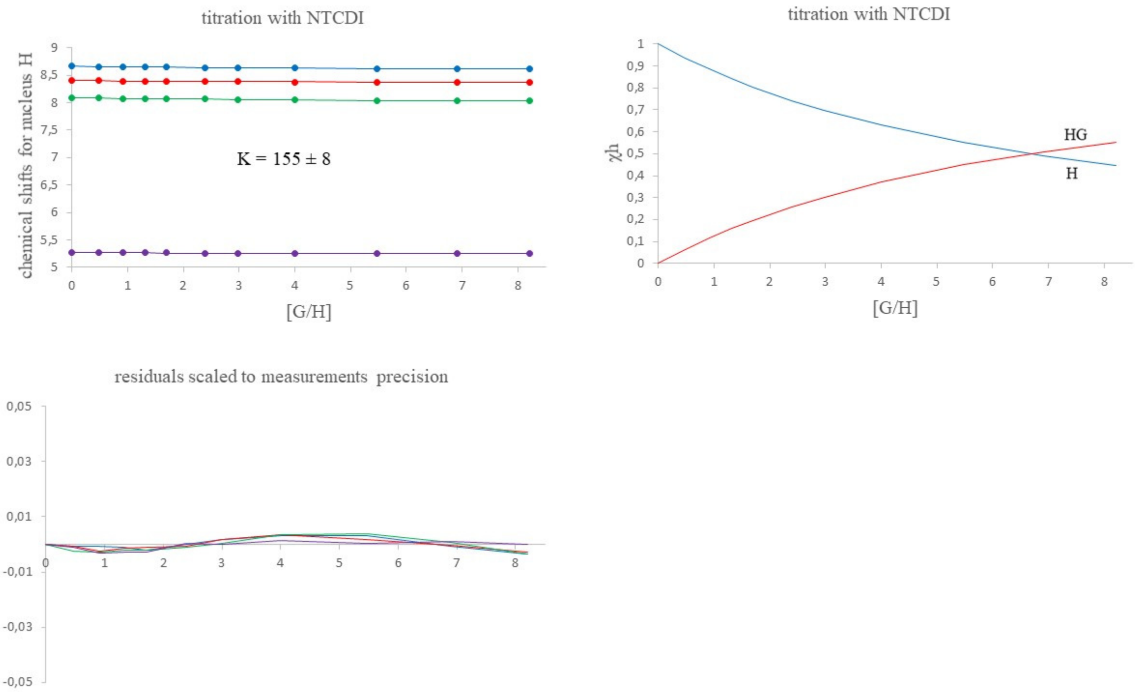

Figure S20. Non-linear least-squares fitting of the chemical shift changes of H during titration experiments of **2** with NTCDI. The Figure on the left represents the speciation profiles.

Titration of 2 with [Pt(C^N^C)(CO)]

Table S8. Data values from the titration study of **2** with [Pt(C^N^C)(CO)]

**[2]** M	**[4]** M	δ_{cH}	δ_{cH}	δ_{cH}	δ_{NCH2}	equiv. **4**
0,00102903	0	8,6608	8,4019	8,2545	5,2692	0
0,00102903	0,000486	8,6586	8,4001	8,2556	5,2659	0,5
0,00102903	0,0009533	8,6555	8,399	8,2513	5,2621	0,9
0,00102903	0,00140297	8,6515	8,3977	8,25	5,2583	1,4
0,00102903	0,00183599	8,6503	8,3964	8,2489	5,2529	1,8
0,00102903	0,00265563	8,6453	8,3945	8,2469	5,2459	2,6
0,00102903	0,00341874	8,6421	8,3927	8,2445	5,2405	3,3
0,00102903	0,00479727	8,6353	8,389	8,2427	5,2313	4,7
0,00102903	0,00708168	8,6237	8,3846	8,239	5,2167	6,9
0,00102903	0,00967254	8,6113	8,3804	8,2334	5,2005	9,4

0,00102903	0,01239294	8,604	8,378	8,229	5,1859	12,0

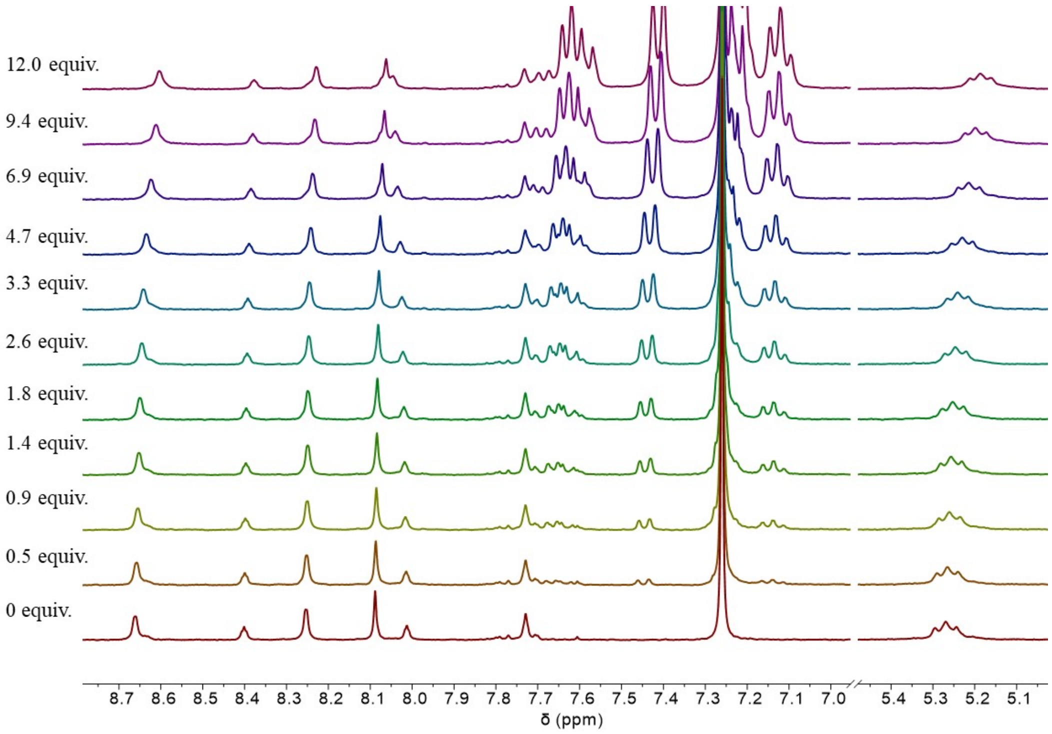

Figure S21. Selected region and spectra of the titration of complex **2** with [Pt(C^N^C)(CO)].

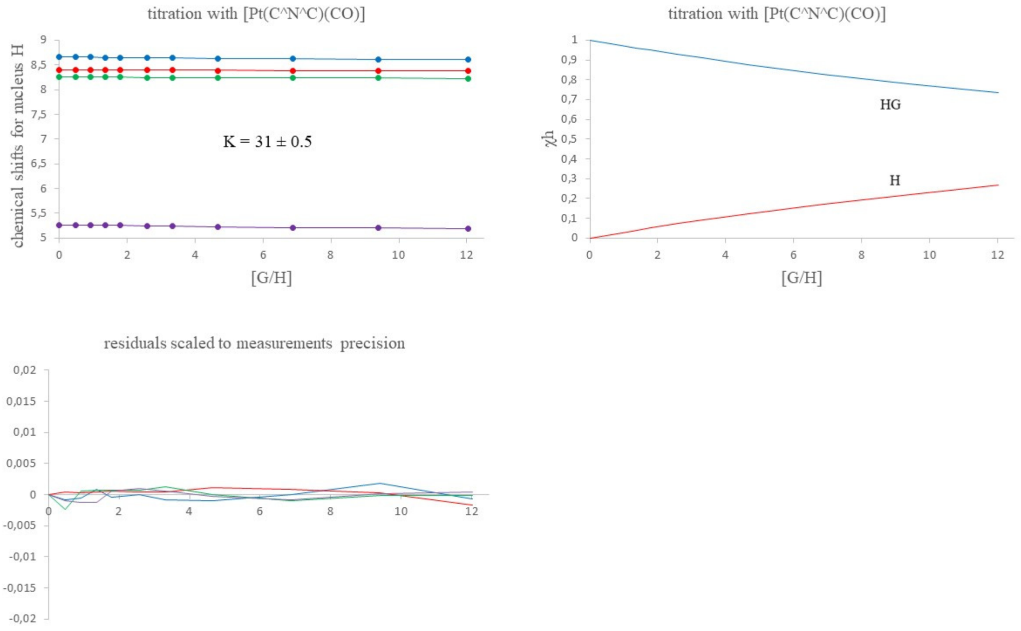

Figure S22. Non-linear least-squares fitting of the chemical shift changes of H during titration experiments of **2** with [Pt(C^N^C)(CO)]. The Figure on the left represents the speciation profiles.

Titration of 2 with [Au(C^N^C)(C≡CC$_6$H$_4$-OCH$_3$-p)].

Table S9. Data values from the titration study of **2** with [Au(C^N^C)(C≡CC$_6$H$_4$-OCH$_3$-*p*)].

**[2]** M	**[3]** M	δc$_H$	δc$_H$	δc$_H$	equiv. **3**
0,00108728	0	8,664	8,4003	8,2537	0
0,00108728	0,00020403	8,6635	8,3994	8,2514	0,2
0,00108728	0,00040022	8,6603	8,3981	8,2505	0,4
0,00108728	0,000589	8,6586	8,3978	8,248	0,5
0,00108728	0,0007708	8,6574	8,3968	8,2466	0,7
0,00108728	0,0011149	8,6556	8,3956	8,2444	1,0
0,00108728	0,00143528	8,6537	8,3949	8,2431	1,3
0,00108728	0,00201402	8,6523	8,3942	8,2409	1,8
0,00108728	0,00297307	8,6502	8,3936	8,2383	2,7
0,00108728	0,00406078	8,6477	8,3923	8,2367	3,7
0,00108728	0,00520288	8,6468	8,3914	8,2348	4,8

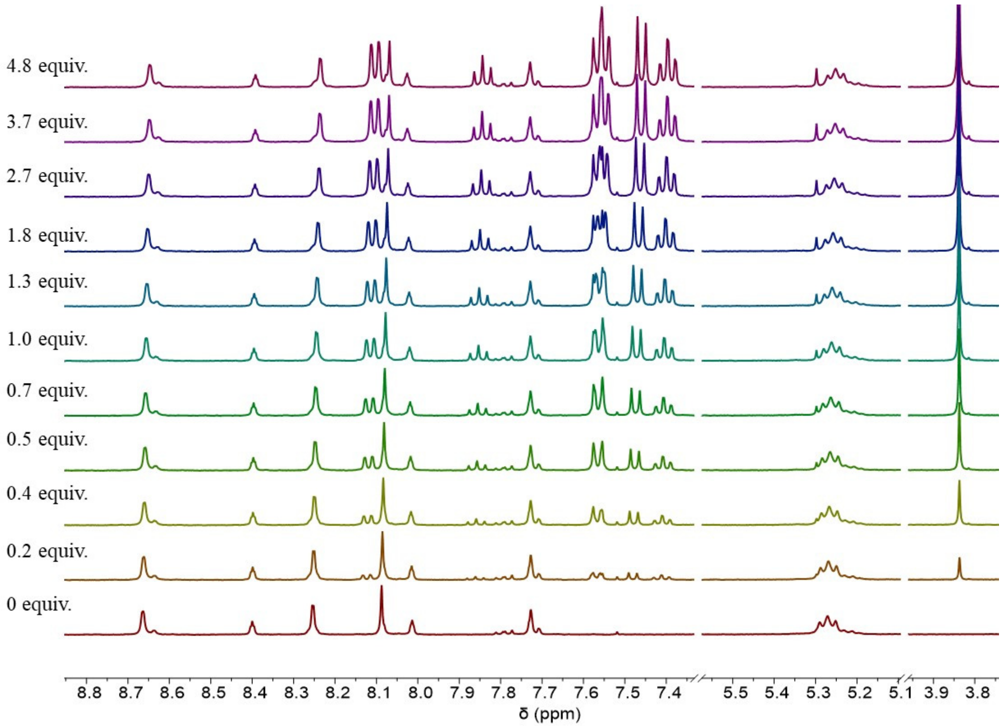

Figure S23. Selected region and spectra of the titration of complex **2** with [Au(C^N^C)(C≡CC$_6$H$_4$-OCH$_3$-*p*)].

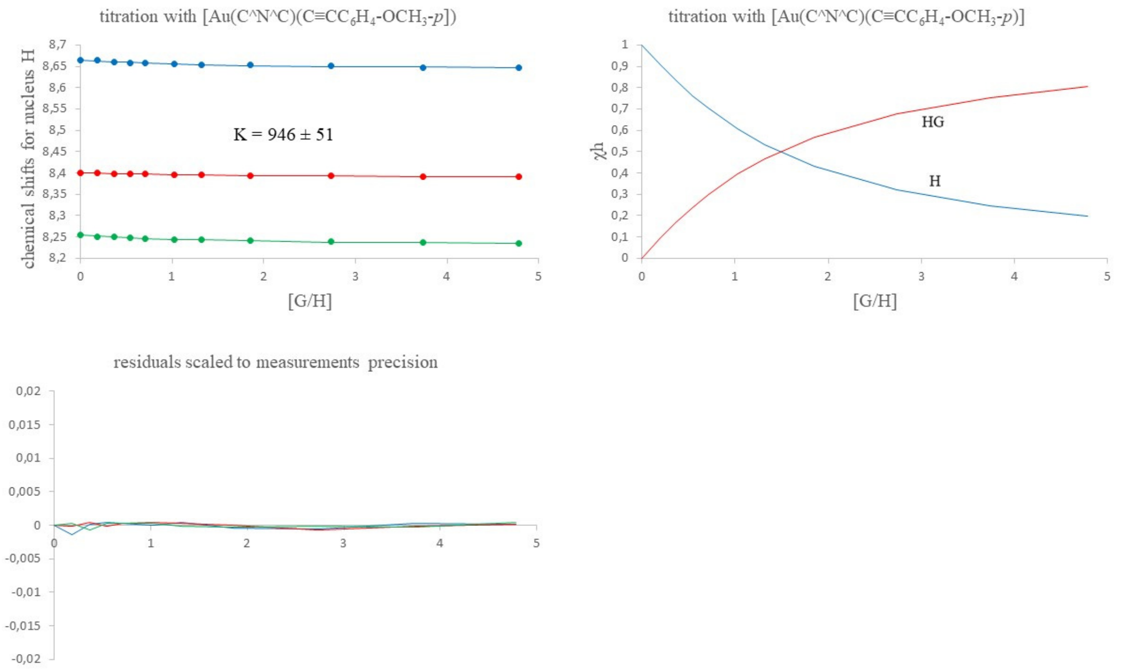

Figure S24. Non-linear least-squares fitting of the chemical shift changes of H during titration experiments of **2** with [Au(C^N^C)(C≡CC_6H_4-OCH_3-*p*)]. The Figure on the left represents the speciation profiles.

Titration of 2 with TNFLU.

Table S10. Data values from the titration study of **2** with TNFLU.

**[2]** M	**[TNFLU]** M	δc_H	δc_H	δc_H	δ_{NH2}	equiv. **TNFLU**
0,00106786	0	8,66	8,4	8,25	5,27	0
0,00106786	0,00075656	8,66	8,4	8,25	5,27	0,7
0,00106786	0,00145709	8,65	8,4	8,25	5,26	1,4
0,00106786	0,00210757	8,65	8,4	8,25	5,26	2,0
0,00106786	0,0027132	8,65	8,39	8,25	5,26	2,5
0,00106786	0,00380723	8,65	8,39	8,24	5,26	3,6
0,00106786	0,00476865	8,64	8,38	8,24	5,25	4,5
0,00106786	0,00601048	8,64	8,38	8,23	5,25	5,6
0,00106786	0,0073765	8,63	8,37	8,23	5,24	6,9
0,00106786	0,00874252	8,62	8,37	8,23	5,24	8,2
0,00106786	0,00983534	8,62	8,37	8,22	5,23	9,2

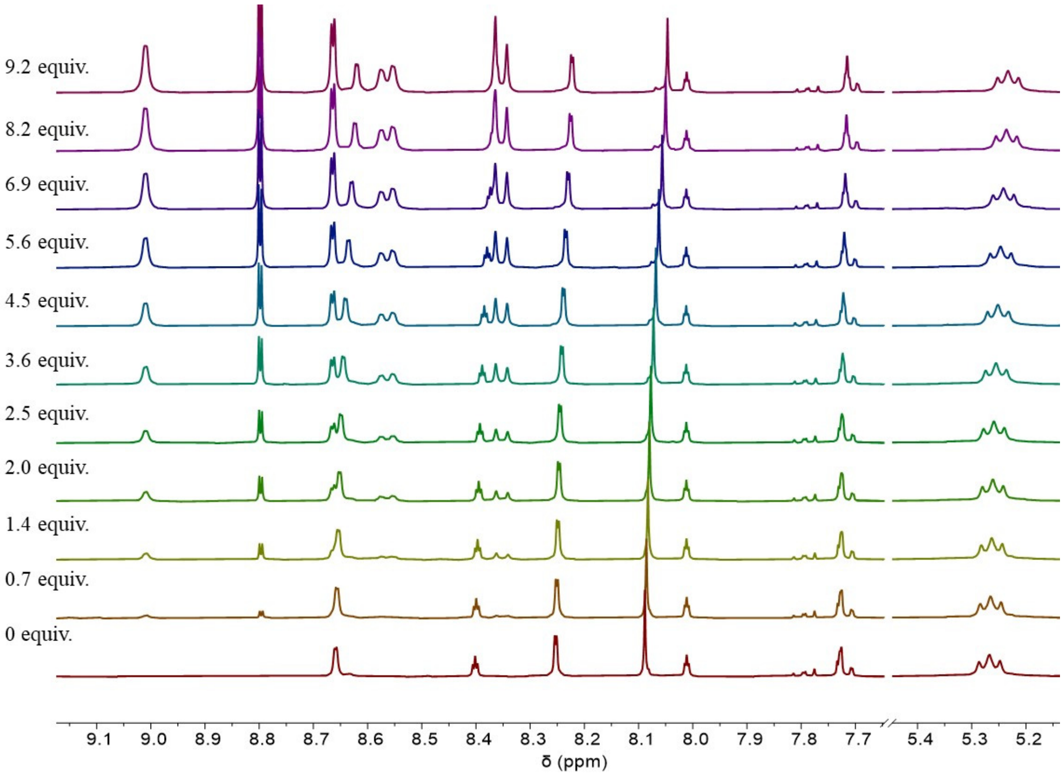

Figure S25. Selected region and spectra of the titration of complex **2** with TNFLU.

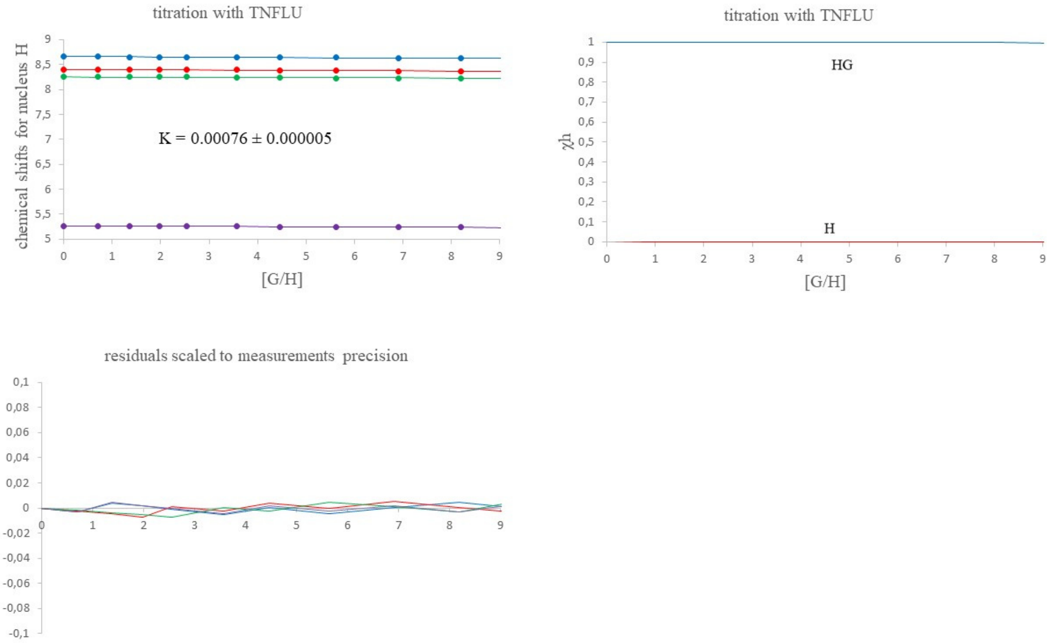

Figure S26. Non-linear least-squares fitting of the chemical shift changes of H during titration experiments of **2** with TNFLU. The Figure on the left represents the speciation profiles.

4. High resolution mass spectra

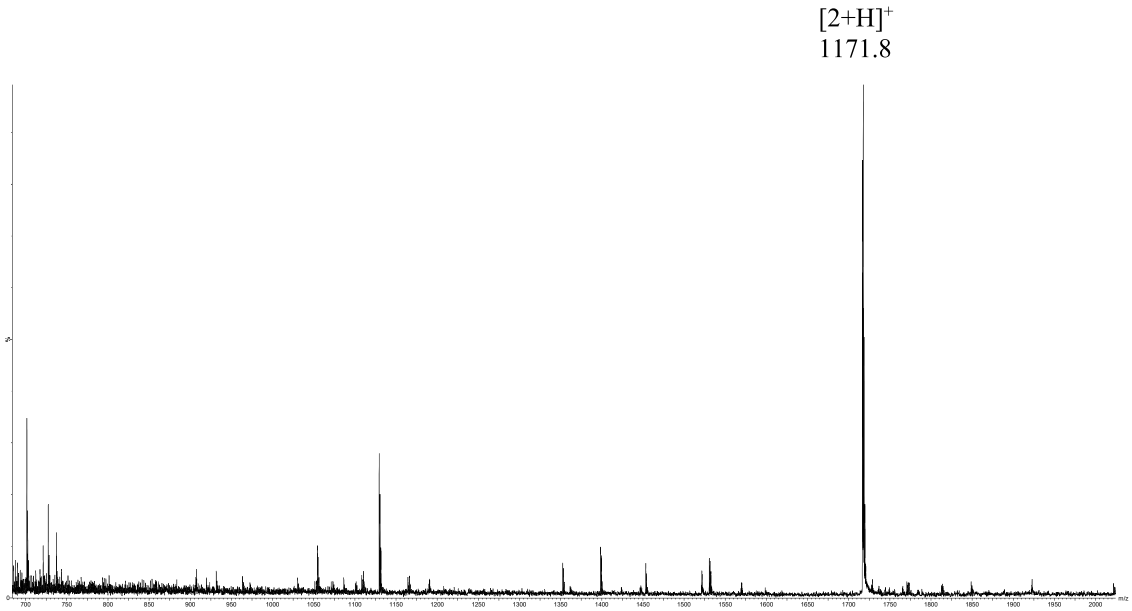

Figure S27. Selected region of the HRMS ESI-TOF-MS (positive mode) spectrum of **2**

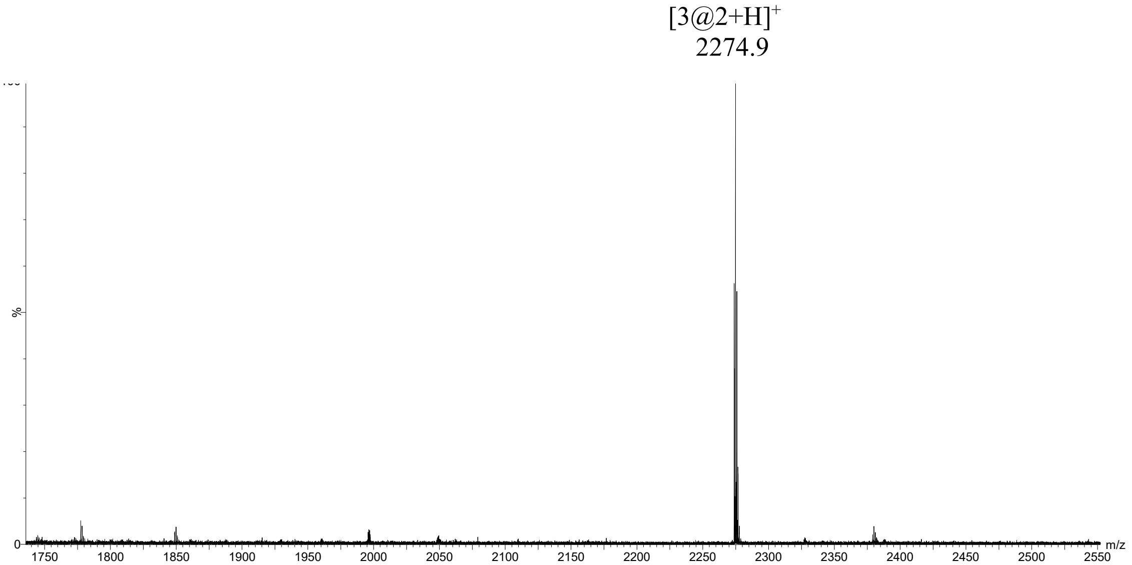

Figure S28. Selected region of the HRMS ESI-TOF-MS (positive mode) spectrum of **3@2**

5. DOSY experiments

The experiments were carried out in $CDCl_3$, at constant concentrations of 5 mM.

Complexes	G (m^2/s)
**2**	5.05 10^{-10}
**3@2**	4.80 10^{-10}

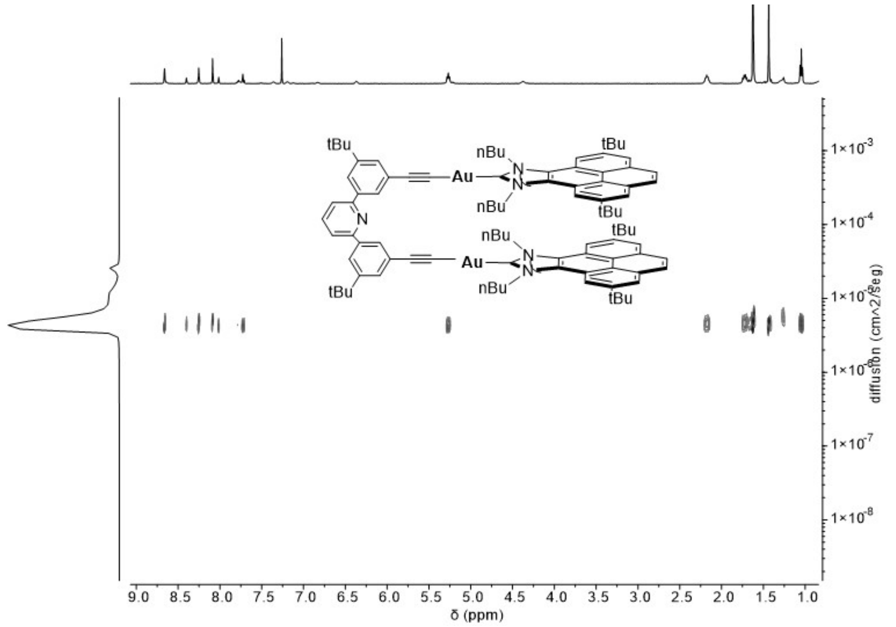

Figure S29. DOSY NMR spectrum of **2**

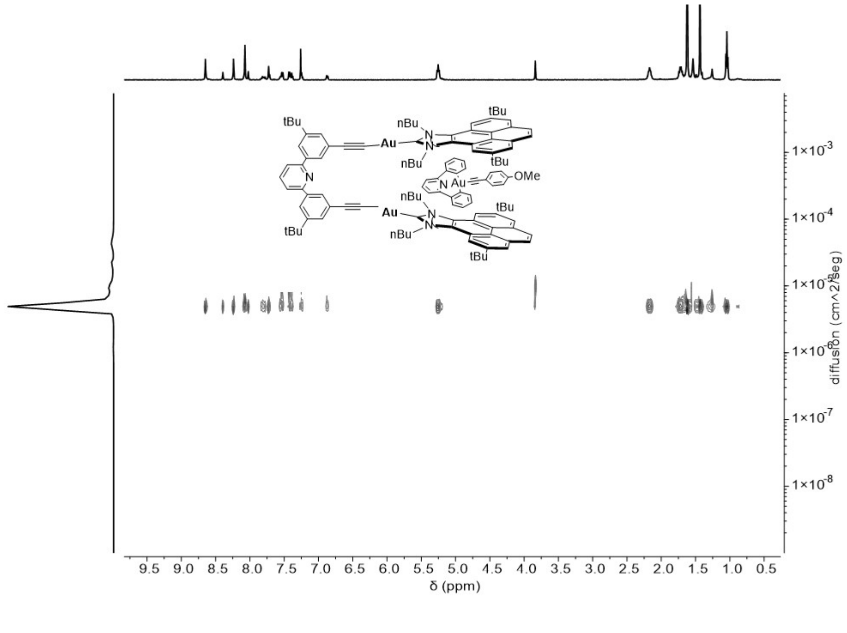

Figure S30. DOSY NMR spectrum of **3@2**

6. IR

The infrared spectra (FTIR) were performed on a FT/IR-6200 (Jasco) spectrometer equipped with a Pro One ATR with a spectral window of 4000-400 cm^{-1}.

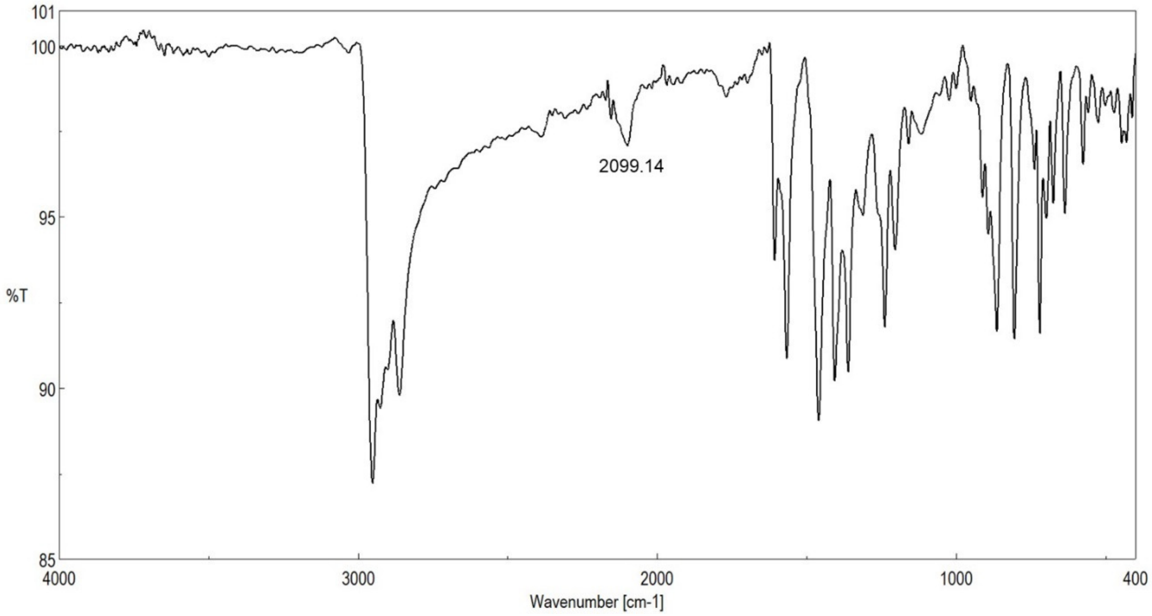

Figure S31. IR spectrum of **2**

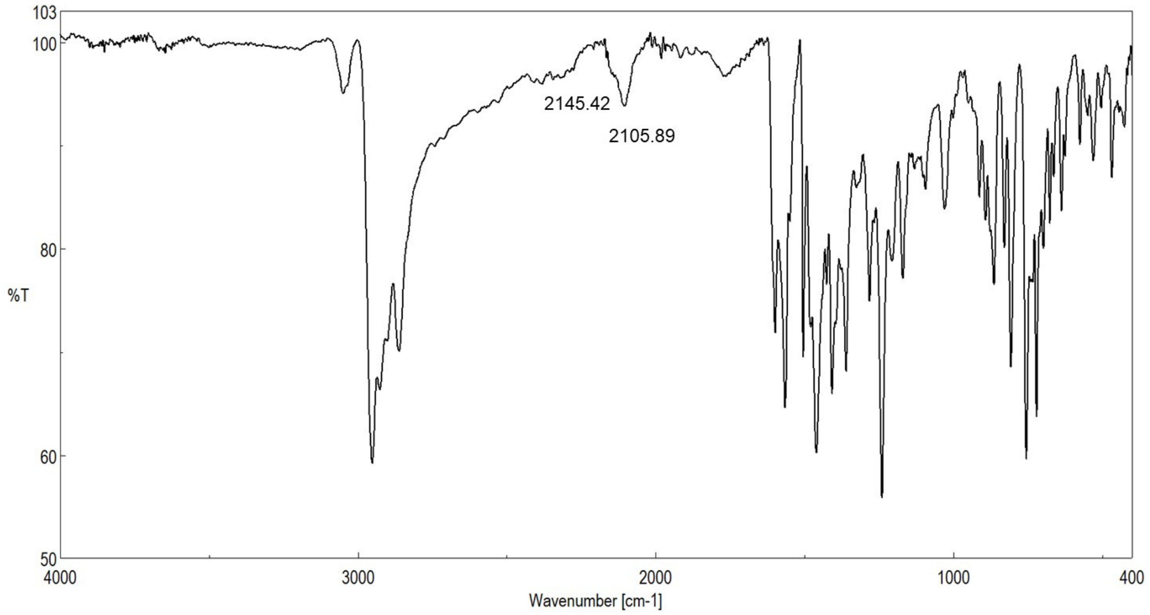

Figure S32. IR spectrum of **3@2**
